# Down-regulation of PDK4 is Critical for the Switch of Carbohydrate Catabolism during Syncytialization of Human Placental Trophoblasts

**DOI:** 10.1038/s41598-017-09163-8

**Published:** 2017-08-16

**Authors:** Xiaohui Liu, Rujuan Zuo, Yirong Bao, Xiaoxian Qu, Kang Sun, Hao Ying

**Affiliations:** 1 Shanghai First Maternity and Infant Hospital, Tongji University School of Medicine, Shanghai, 201204 P. R. China; 2 Center for Reproductive Medicine, Ren Ji Hospital, School of Medicine, Shanghai Jiao Tong University, Shanghai, 200135 P. R. China; 3 Shanghai Key Laboratory for Assisted Reproduction and Reproductive Genetics, Shanghai, 200135 P. R. China

## Abstract

Pyruvate dehydrogenase kinase (PDK) is known as a gatekeeper directing the carbon flux into glycolysis via inhibition of the pyruvate dehydrogenase complex. During syncytialization of placental trophoblasts, both ATP production and oxygen consumption are increased to meet enhanced energetic demands by syntiotrophoblasts. We hypothesized that down-regulation of PDK expression may play a central role in the switch from glycolysis to oxidative phosphorylation (OXPHOS) during syncytialization. By using primary human trophoblasts, we demonstrated that PDK4 was the dominating PDK isoform in human cytotrophoblasts, and its abundance was substantially decreased upon syncytialization, which was accompanied by decreases in lactate production and increases in ATP production. Knock-down of PDK4 expression reduced lactate production and increased ATP production, while over-expression of PDK4 increased lactate production and decreased ATP production, indicating that down-regulation of PDK4 is key to the shift from glycolysis to OXPHOS during syncytialization. Moreover, human chorionic gonadotropin (hCG)/cAMP/PKA pathway was demonstrated to be involved in the down-regulation of PDK4 expression upon syncytialization. Taken together, our findings disclosed that down-regulation of PDK4 is critical for the metabolic shift from glycolysis to OXPHOS during syncytialization, which may be a prerequisite for the proper implementation of syncytiotrophoblast functions.

## Introduction

Carbohydrate metabolism is well-known as the most fundamental resource for biomass and bioenergy. Two distinctive carbohydrate catabolic pathways, glycolysis and oxidative phosphorylation (OXPHOS), are alternatively utilized by mammalian cells. In OXPHOS, the intermediary metabolite pyruvate is fated to be oxidized to acetyl-CoA by the pyruvate dehydrogenase (PDH) complex in the mitochondria. Acetyl-CoA enters tricarboxylic acid (TCA) cycle, which is coupled with the electron transporting respiratory system for abundant ATP production^1^. Under conditions of anaerobic and aerobic glycolysis (Warburg effect), due to inactivation of the PDH complex, pyruvate is alternatively fermented into lactate by lactate dehydrogenase (LDH)^2^. Since glycolysis can balance the demands between biosynthesis by accumulating intermediary metabolites and bioenergetics by producing moderate amount of ATP molecules, it is favored by many high proliferative cells such as certain tumor and stem cells^2–4^. However, a metabolic transition from glycolysis to OXPHOS can occur once these cells undergo differentiation since OXPHOS provides adequate ATP to meet the increased energy demands for the differentiated cells^4^.

Given that the catalytic activity of PDH complex is decisive for OXPHOS, pyruvate dehydrogenase kinase (PDK), which inactivates the PDH complex by phosphorylating its E1α subunit (PDHE1α), is believed to be a critical gatekeeper directing carbon flux into glycolysis from OXPHOS^1, 5^. There are four documented isoforms of PDK (PDK1, 2, 3 and 4) which are encoded by four distinct genes^6^. It has been reported that glycolysis maintained by PDK2 and PDK4 is critical for the pluripotency of hematopoietic stem cells while down-regulations of PDK2 and PDK4 expression and the consequent transition from glycolysis to OXPHOS are required for the differentiation of these cells^7^.

Of interest, human placental villous cytotrophoblast cells share many characteristics with tumor and stem cells, possessing the capability of differentiating into non-proliferative multi-nucleated syncytiotrophoblasts^8^. By covering the chorionic villi, syncytiotrophoblasts not only form the front line of nutrient exchange interface between mother and fetus but are also the major site of placental hormone synthesis. These fundamental changes in proliferative and functional properties in trophoblasts during syncytialization highly suggest that the pathway of carbohydrate catabolism may be altered in a similar way as in tumor and stem cells. Previous study has demonstrated that both ATP production and oxygen consumption are increased during syncytialization of human placental trophoblasts^9^. Given that OXPHOS consumes abundant oxygen and produces more ATP molecules, we hypothesized that the carbohydrate catabolism might shift from glycolysis to OXPHOS during syncytialization to meet the enhanced energetic demands to accomplish the sophisticated functions of the syncytiotrophoblasts, and this transition might be a consequence of down-regulation of PDK expression upon syncytialization. Here, we examined this hypothesis in an *in vitro* model of syncytialization of primary human placental trophoblasts^10, 11^.

## Results

### Metabolic shift from glycolysis to OXPHOS during syncytialization

**Figure 1:**
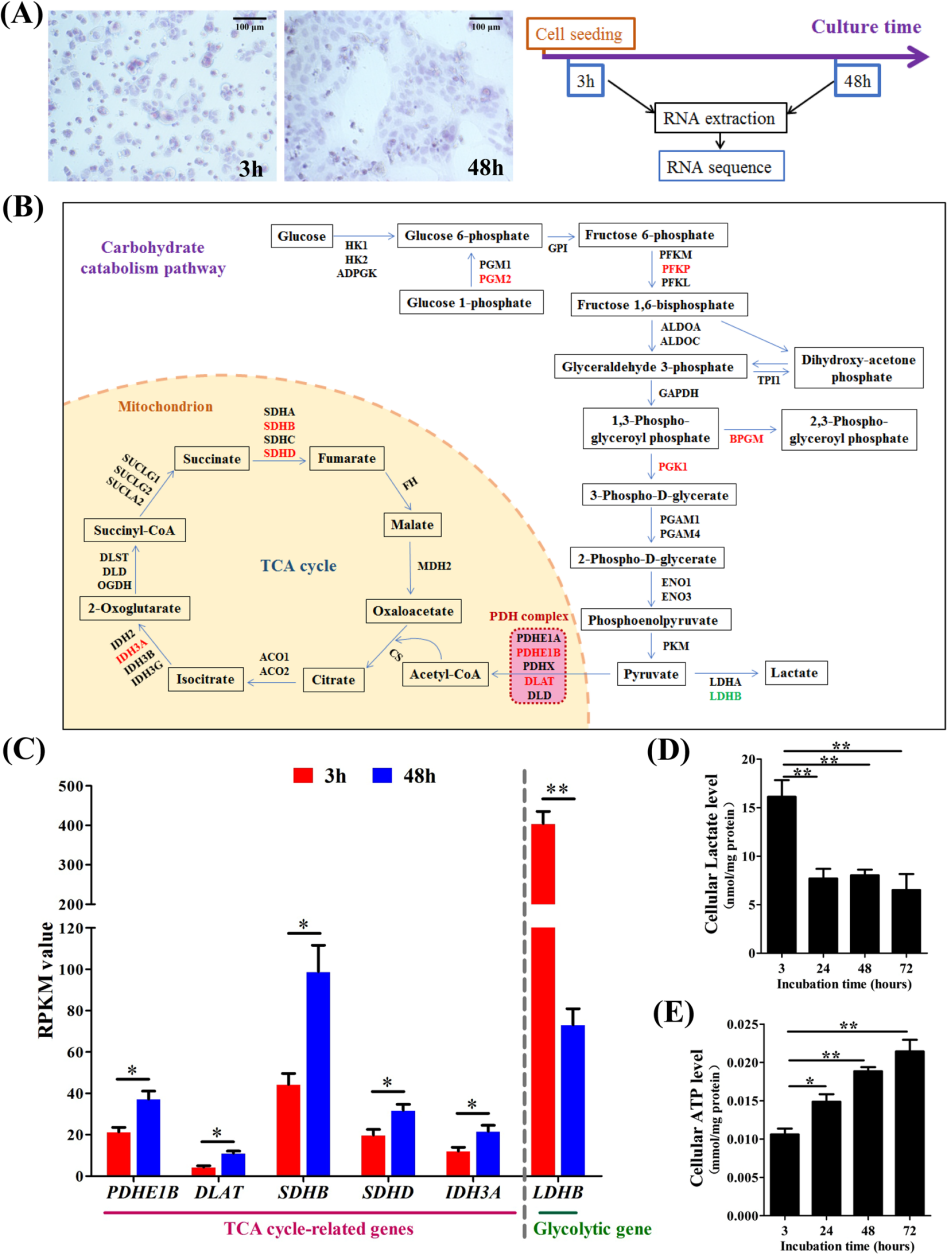
Alteration of carbohydrate catabolic pathway during syncytialization of human placental trophoblasts. (**A**) Hematoxylin staining showing morphological changes of human placental trophoblasts during syncytialization. The trophoblasts were collected before (3 h) and after (48 h) syncytialization for transcriptomic analyses. (**B**) A diagram depicting the changes of genes encoding enzymes involved in glycolysis and tricarboxylic acid (TCA) cycle during syncytialization with transcriptomic analyses. Red: up-regulated genes, Green: down-regulated gene. (**C**) Changes in the values of Reads per Kilobases per Millionreads (RPKM) of the differentially expressed genes during syncytialization. n = 3. (**D**) and (**E**) Changes in the cellular lactate (n = 5, **D**) and ATP levels (n = 3, **E**) during syncytialization. *P < 0.05; **P < 0.01 against 3 hours.

To obtain a transcriptional map of carbohydrate catabolism during syncytialization, we isolated primary human trophoblast cells from term placenta, which undergo spontaneous syncytialization under the culture condition with 10% fetal bovine serum (FBS), and then sequenced the transcriptomes of these cells before (3 hours after plating) and after (48 hours after plating) syncytialization with particular attention to carbohydrate catabolism (Fig. 1A)^10, 11^. The differential expression analysis identified changes of several genes involved in carbohydrate catabolism during syncytialization (Fig. 1B and Table S1). The Reads per Kilobases per Millionreads (RPKM) values for 5 OXPHOS-related genes (*PDHE1B, DLAT, SDHB, SDHD* and *IDH3A*) were significantly up-regulated whereas a glycolytic gene *LDHB* was significantly down-regulated during syncytialization (Fig. 1B and C and Table S1). These changes in gene expression were accompanied with decreases in lactate level (Fig. 1D) and increases in ATP production during syncytialization (Fig. 1E). These data suggest that a metabolic shift from glycolysis to OXPHOS occurs during syncytialization.

### Down-regulation of PDK4 abundance during syncytialization

**Figure 2:**
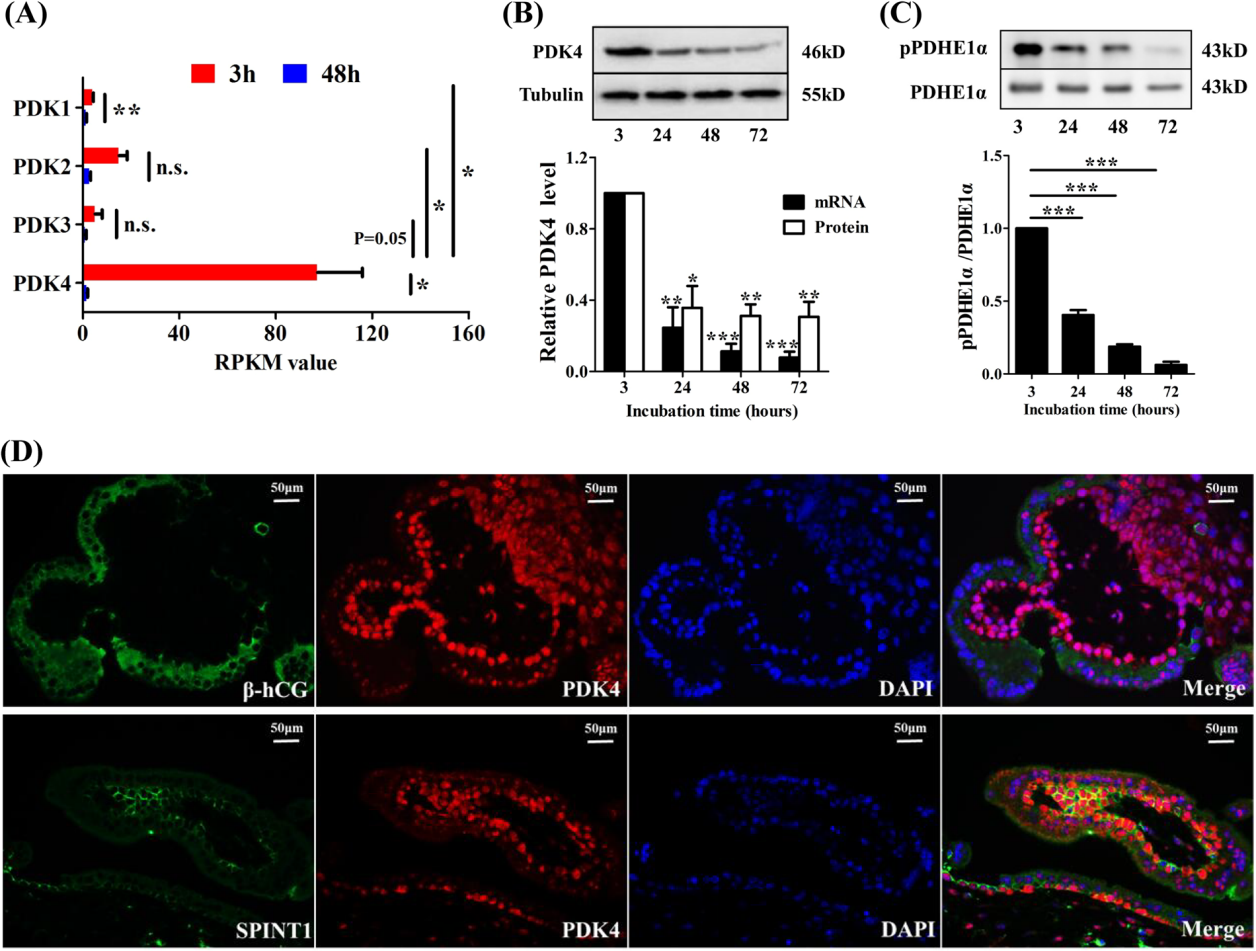
PDK4 expression during syncytialization of human placental trophoblasts. (**A**) Changes of the RPKM values for PDK family members in trophoblasts before (red column, 3 hours) and after (blue column, 48 hours) syncytialization. (**B**) Changes in PDK4 mRNA (black column, n = 4) and protein (white column, n = 4) abundance during syncytialization. (**C**) Changes in the phosphorylation of PDHE1α during syncytialization n = 4. (**D**) Representative images showing intense staining of PDK4 (red) in the cytotrophoblast layer and weak staining of PDK4 in the syncytial layer of human chorionic villi at early gestation. The syncytial and cytotrophoblast layers were labeled with β-hCG (green) and SPINT1 (green) respectively. Nuclei were counterstained with DAPI (blue). n = 3; *P < 0.05; **P < 0.01; ***P < 0.001 against 3 hours; n.s., not significant.

Although the transcriptomic analysis revealed that several OXPHOS-related genes were up-regulated and one glycolytic gene was down-regulated during syncytialization, the catalytic activity of the PDH complex that gates the carbon flux into OXPHOS from glycolysis is of prime importance. Since PDK inactivates PDH by phosphorylating the E1α subunit of PDH complex^1, 5^, it is logical to examine whether the alteration in PDK expression plays a critical role in the metabolic shift from glycolysis to OXPHOS during syncytialization. Transcriptomic analyses revealed that the RPKM value for PDK4 is much higher than that of any of the other three PDK family members in the trophoblasts before syncytialization (Fig. 2A and Table S1), indicating that PDK4 is the dominant isoform of the PDK family in trophoblasts, although other members of PDK family were also detected. The RPKM values for all PDK isoforms were decreased during syncytialization, but the most dramatic reduction was observed with PDK4 (Fig. 2A). Quantitative real time PCR (qRT-PCR) and western blotting analyses confirmed the down-regulation of PDK4 at both mRNA and protein levels during syncytialization (Fig. 2B). Consistently, immunofluorescent staining of human chorionic villous tissue obtained from early pregnancy (6–8 weeks) showed that PDK4 distributed mainly in the cytotrophoblasts which were also stained for SPINT1, a marker for cytotrophoblasts^12^, while the syncytiotrophoblasts, which were identified with staining for the β subunit of human chorionic gonadotropin (β-hCG)^10^, were stained faintly for PDK4 (Fig. 2D). Since PDK4 inhibits the activity of PDH complex by phosphorylating PDHE1α at serine^293^ ^6^, we assessed the abundance of phosphorylated E1α of PDH (pPDHE1α) at serine^293^ during syncytialization. The abundance of pPDHE1α was substantially reduced in the absence of any changes in the total PDHE1α protein abundance during syncytialization (Fig. 2C). Collectively, these data suggest that PDK4 is the dominant PDK isoform expressed in placental trophoblast cells and PDK4 expression is dramatically down-regulated along with decreases in PDHE1α phosphorylation during syncytialization.

### Role of PDK4 in the switch from glycolysis to OXPHOS during syncytialization

**Figure 3:**
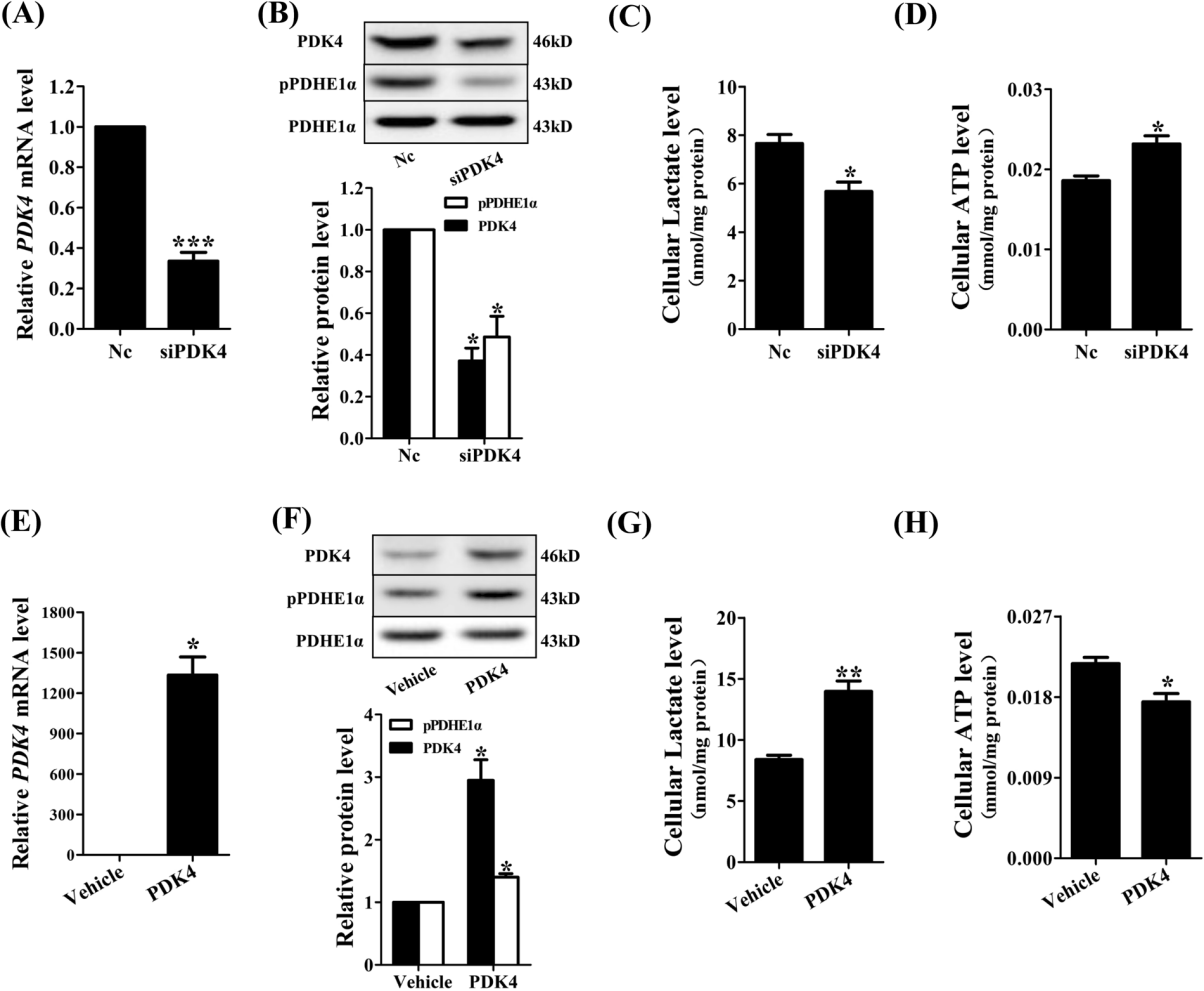
Role of PDK4 in the switch of carbohydrate catabolism in human placental trophoblasts. (**A**) siRNA-mediated knock-down of PDK4 (siPDK4) for 48 hours significantly decreased PDK4 mRNA abundance. n = 6. (**B**) Knock-down of PDK4 (siPDK4) for 48 hours significantly reduced PDHE1α phosphorylation. n = 3. (**C)** and (**D**) siRNA-mediated knock-down of PDK4 decreased cellular lactate levels (n = 5, **C**) while increased cellular ATP levels (n = 5, **D**). Randomly scrambled siRNA served as negative control (Nc). **(E)** Over-expression of PDK4 by transfecting PDK4 plasmid significantly improved PDK4 mRNA abundance. n = 3. **(F)** Over-expression of PDK4 significantly increased PDHE1α phosphorylation. n = 3. (**G**) and **(H**) Over-expression of PDK4 increased lactate levels (n = 5, **G**) while decreased cellular ATP levels (n = 5, **H**). *P < 0.05; **P < 0.01 against Nc (**A** to **D**) or Vehicle (**E** to **H**).

To assess whether PDK4 indeed plays a role in the metabolic switch from glycolysis to OXPHOS during syncytialization, isolated cytotrophoblasts were transfected with small interfering RNA (siRNA) targeting PDK4 or constructed plasmid expressing PDK4. Small interfering RNA-mediated knock-down of PDK4 expression significantly decreased PDHE1α phosphorylation (Fig. 3A and B) and cellular lactate levels (Fig. 3C) while increased cellular ATP levels (Fig. 3D). Conversely, over-expression of PDK4 increased PDHE1α phosphorylation (Fig. 3E and F) and lactate levels (Fig. 3G) while decreased ATP levels (Fig. 3H). These data indicate that down-regulation of PDK4 is involved in the switch of carbohydrate catabolism from glycolysis to OXPHOS during syncytialization.

### Role of PDK4 in syncytiotrophoblast functions

**Figure 4:**
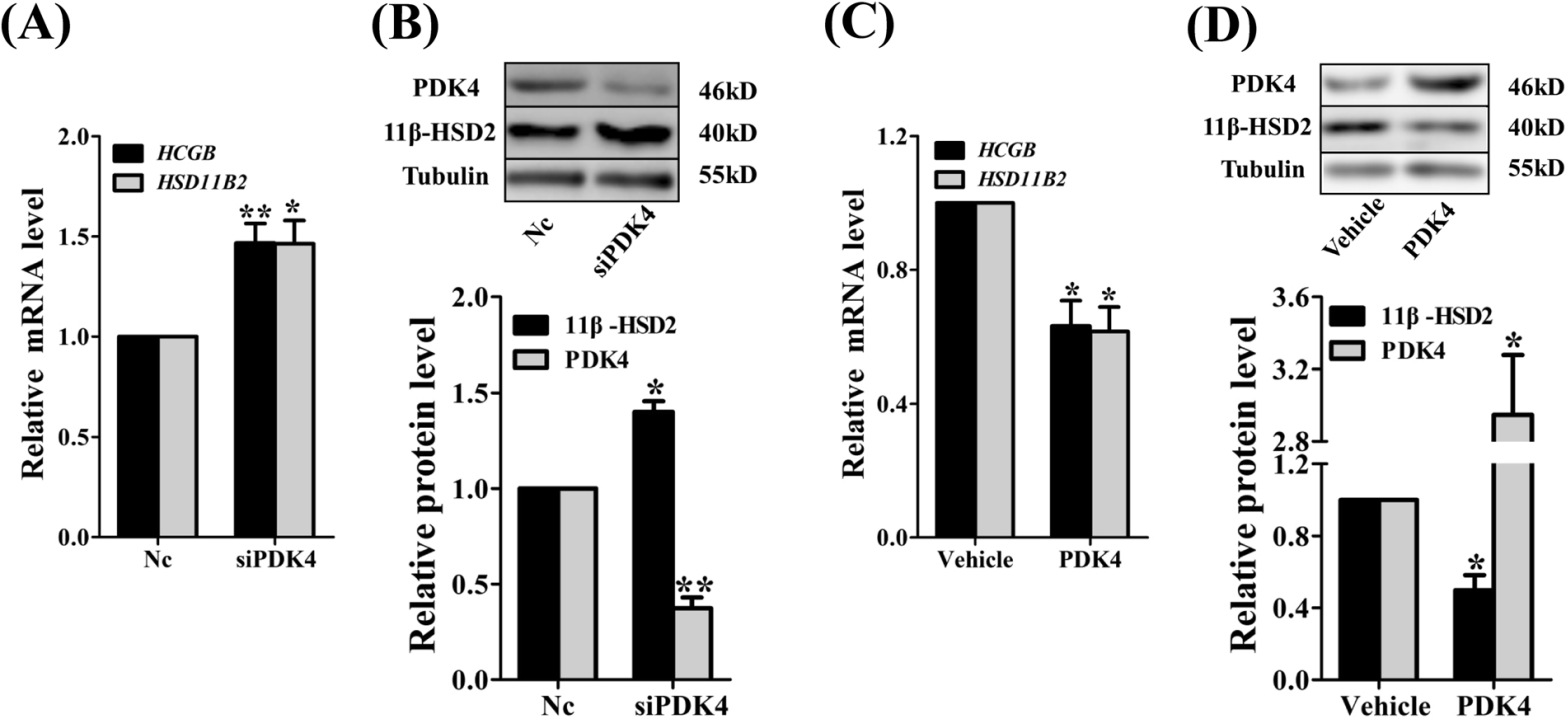
Role of PDK4 in syncytiotrophoblast functions. (**A**) and (**B**) The abundance of 11β-HSD2 mRNA (n = 4, **A**) and protein (n = 3, **B**) and β-hCG mRNA (n = 4, **A**) was significantly increased by siRNA-mediated knock-down of PDK4 expression. Randomly scrambled siRNA served as negative control (Nc). **(C)** and (**D**) Over-expression of PDK4 significantly decreased the abundance of 11β-HSD2 mRNA (n = 4, **C**) and protein (n = 3, **D**) and β-hCG mRNA (n = 4, **C**). *P < 0.05; **P < 0.01; ***P < 0.001 against Nc (**A** and **B**) or Vehicle (**C** and **D**).

To examine whether manipulating PDK4 expression affects physiological functions of syncytiotrophoblasts, we measured the expression of β-hCG, a crucial hormone in pregnancy maintenance^13^, and the expression of 11β-hydroxysteroid dehydrogenase type 2 (11β-HSD2), an enzyme that functions as the placental glucocorticoid barrier by inactivating biologically-active cortisol to its inactive counterpart cortisone^14^. Knock-down of PDK4 expression increased the abundance of β-hCG and 11β-HSD2 (Fig. 4A and B), whereas over-expression of PDK4 decreased the abundance of β-hCG and 11β-HSD2 (Fig. 4C and D). These data indicate that the shift of carbohydrate catabolism from glycolysis to OXPHOS as a result of down-regulation of PDK4 is implicated in the proper implementation of syncytiotrophoblast functions as exemplified by β-hCG and 11β-HSD2 expression in this study.

### Role of hCG/cAMP/PKA pathway in the regulation of PDK4 expression during syncytialization

**Figure 5:**
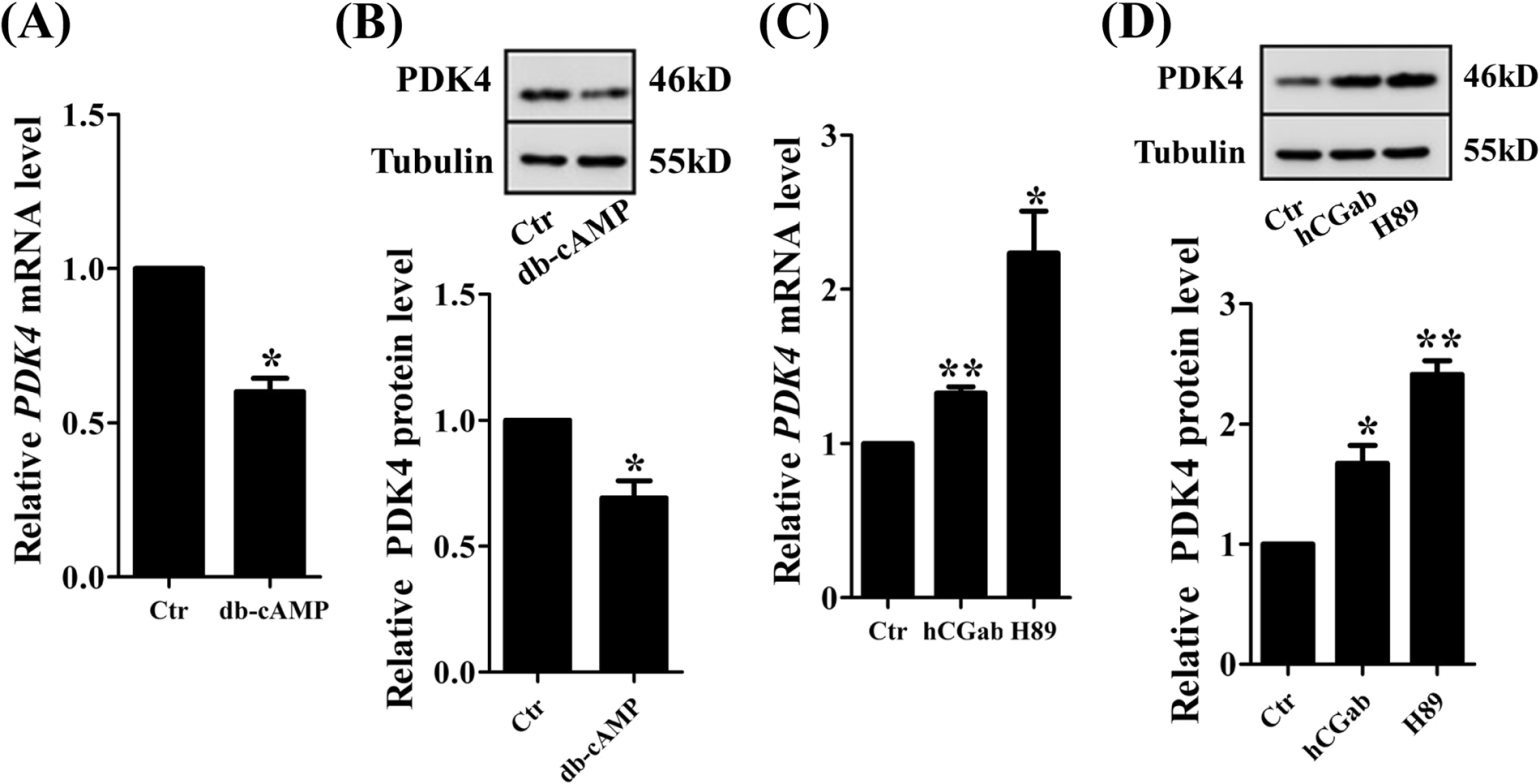
Role of activation of the cAMP/PKA signaling pathway by hCG in the regulation of PDK4 expression in human placental trophoblasts. (**A**) and (**B**) Treatment of trophoblasts with dibutyryl-cAMP (db-cAMP, 200 μM) for 24 hours significantly decreased the amounts of PDK4 mRNA (n = 6, **A**) and protein (n = 3, **B**). (**C**) and (**D**) Treatment of trophoblasts with β-hCG antibody (hCGab, 1:100) or PKA inhibitor H89 (20 μM) for 24 hours significantly increased the abundance of PDK4 mRNA (n = 4, **C**) and protein (n = 3, **D**). *P < 0.05; **P < 0.01 against Ctr.

Among the sophisticated molecular pathways that regulate syncytialization, the cAMP/PKA pathway activated by hCG is well recognized^15, 16^. In addition, the cAMP/PKA pathway is also known to be linked to carbohydrate metabolism in a number of cell types^17, 18^. Thus, we postulated that activation of the cAMP/PKA pathway by hCG during syncytialization might be responsible for the down-regulation of PDK4 expression. Our data showed that the cAMP analogue dibutyryl-cAMP (db-cAMP, 200 μM) significantly decreased the abundance of PDK4 mRNA (Fig. 5A) and protein (Fig. 5B), while inhibiting PKA with H89 (20 μM) or neutralizing hCG with an antibody against β-hCG (1:100) significantly increased the abundance of PDK4 mRNA (Fig. 5C) and protein (Fig. 5D). These results suggest that activation of hCG/cAMP/PKA pathway participates in the down-regulation of PDK4 expression during syncytialization.

## Discussion

In this study, we have demonstrated that PDK4 is the dominant PDK isoform in human placental trophoblasts and its abundance is substantially decreased along with a reduction in PDHE1α phosphorylation during syncytialization. Considering that there are concurrent up-regulation of OXPHOS-related genes and down-regulation of a glycolysis-related gene as well as decreases in lactate production and increases in ATP production during syncytialization, we believe that a shift from glycolysis to OXPHOS in carbohydrate catabolism occurs in trophoblasts during syncytialization and the down-regulation of PDK4 expression plays a central role in directing carbon flux into OXPHOS from glycolysis. The crucial role of PDK4 in this switch is endorsed by reciprocal changes in the production of lactate and ATP upon siRNA-mediated knocking-down of PDK4 and over-expressing PDK4. However, it should be noted that other PDK members may also contribute to the metabolic shift since their abundance is reduced during syncytialization as well. However, we believe that PDK4 plays a dominant role in this switch because the abundance of the other PDK isozymes is much less than PDK4 and their changes during syncytialization are also much smaller than PDK4. Moreover, manipulation of PDK4 expression significantly changed the carbohydrate catabolism. Once the carbon flux is directed into OXPHOS as a result of down-regulation of PDK4, enzymes in TCA cycle which are up-regulated upon syncytialization can thus ensure efficient OXPHOS.

It is known that the cAMP signaling pathway is involved in both syncytialization and carbohydrate metabolism^16, 17^. Previous studies have demonstrated that activation of the cAMP pathway is a determinant of carbohydrate metabolic plasticity via up-regulation of OXPHOS-associated enzymes and enhancement of mitochondria respiration in a number of cell types^17, 19^. Although PKA can directly phosphorylate a number of mitochondrial enzymes involved in OXPHOS^17, 20^, PKA has been demonstrated to be incapable of phosphorylating PDHE1α^21^. This study disclosed that activation of the hCG/cAMP/PKA pathway is a causative factor for the down-regulation of PDK4 expression during syncytialization. Given the role of PDK4 versus hCG/cAMP/PKA in regulating each other, we believe that there might be a feed-forward relation between the downregulation of PDK4 expression and the accumulation of hCG production during syncytializaiton. However, at the current stage, we are unclear whether this down-regulation of PDK4 by hCG/cAMP/PKA pathway is secondary to syncytialization or a direct effect on it expression.

Accumulating evidence indicates that the status of cell differentiation is closely correlated with carbohydrate catabolic pathway and mitochondrial activity. Highly proliferative stem cells usually rely on glycolysis and have few mitochondria, while differentiating stem cells normally have an evident metabolic shift to OXPHOS^22^. Induction of carbohydrate catabolism toward glycolysis can compromise the differentiation of stem cells^22–25^. Likewise, PDK2 and PDK4, as the gatekeepers for carbohydrate catabolic pathways, have been shown to play a critical role in the transition from proliferation to differentiation of the hematopoietic stem cells^7^. Therefore, glycolysis is considered to be an indicator of cell pluripotency and poor differentiation^22^. Placental cytotrophoblast cells behave in many ways like the progenitor cells, and they are capable of differentiating into terminally differentiated syncytiotrophoblast cells^13^. Our findings that cytotrophoblasts are glycolytic are in line with their pluripotent properties, while the findings that syncytiotrophoblasts adopt OXPHOS in carbohydrate catabolism are consistent with the loss of proliferative ability. These findings endorsed a previous study showing that the mitochondrial activity is repressed in cytotrophoblasts but enhanced in syncytiotrophoblasts^26^. In addition, the adoption of glycolysis in cytotrophoblasts can provide necessary intermediate metabolites required for proliferation. Therefore, we believe that the adoption of glycolysis in cytotrophoblasts is essential to keep their proliferative ability, while the shift from glycolysis to OXPHOS during syncytialization is not only a property of terminally differentiated syncytiotrophoblats, but can also produce adequate ATP to meet the enhanced energy demands by syncytiotrophoblasts for their sophisticated functions. Our findings that over-expression of PDK4 significantly jeopardized the expression of β-hCG and 11β-HSD2 in syncytiotrophoblasts indicate a crucial role of the switch of carbohydrate catabolism to OXPHOS in the establishment of these functions.

**Figure 6:**
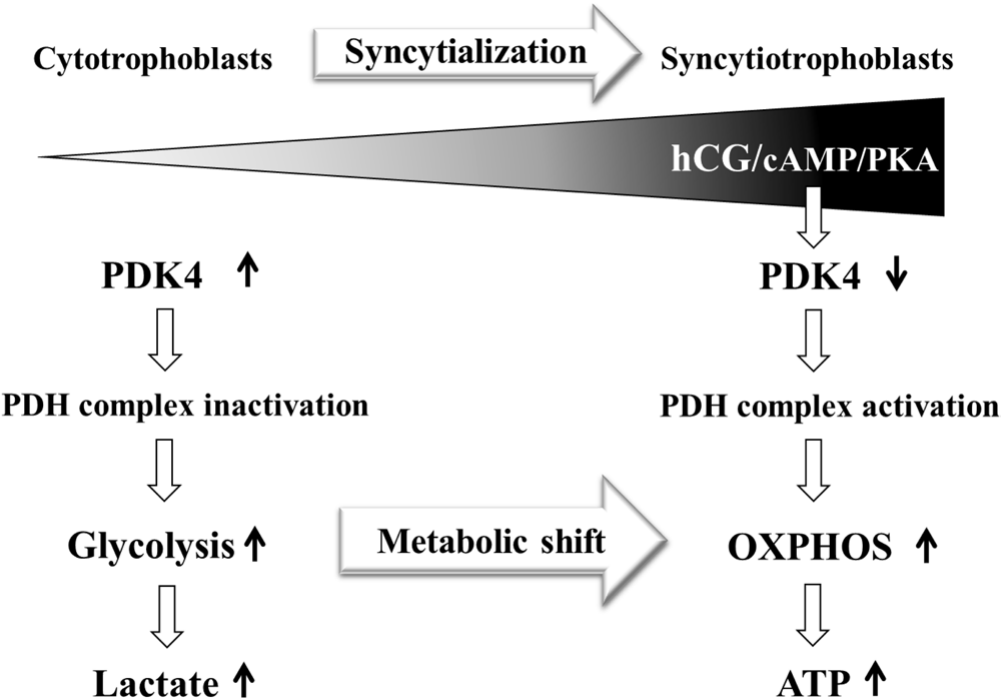
Diagram illustrating the switch from glycolysis to OXPHOS via down-regulation of PDK4 expression upon activation of the cAMP pathway by hCG during syncytialization of the trophoblasts.

In conclusion, we have demonstrated in this study that PDK4 is the dominant PDK isoform in human placental trophoblasts, and down-regulation of PDK4 expression upon activation of the hCG/cAMP/PKA pathway is critical for the switch of carbohydrate catabolism from glycolysis to OXPHOS during syncytialization, which may implicate in the establishment of syncytial functions (Fig. 6).

## Methods

### Collection of human placental villous tissues

Human placental villous tissues were obtained from uncomplicated early gestations (6–8 weeks) after painless induced-abortion and from term (38–40 weeks) pregnancies after elective cesarean section with written informed consents under a protocol approved by the Ethics Committee of Ren Ji Hospital, School of Medicine, Shanghai Jiao Tong University. All methods were performed in accordance with the relevant guidelines and regulations. The chorionic villi from early gestation were fixed with formalin for immunochemical staining, while the fresh villous tissue from term gestation was processed for cytotrophoblast isolation.

### Immunohistochemical staining

Immunohistochemical staining was performed in chorionic villous sections (n = 5) as previously described^27^. Briefly, after deparaffination and rehydration, the sections were boiled in 10 mM sodium citrate buffer for antigen retrieval and permeablized in PBS containing 0.4% Triton X-100. After blocking with goat serum and subsequent incubation with primary antibodies (Table 1), the sections were incubated with Alexa Fluor 488- or Alexa Fluor 594-labeled secondary antibodies (Proteintech, Rosemont, IL). Nuclei were stained with DAPI (1 μg/mL). The staining signals were examined under a fluorescence microscope (Zeiss, Germany).

**Table 1:** Information of the antibodies for immunohistochemical staining and western blotting.

Target protein	Company	Catalog No.	Applications
PDK4	Proteintech	12949-1-AP	WB (1:1000), IF (1:200)
β-hCG	Thermo Fisher Scientific	MA5-14375	IF (1:100)
SPINT1	Santa Cruz	sc-137159	IF (1:200)
11β-HSD2	Santa Cruz	sc-20176	WB (1:2000)
α-Tubulin	Proteintech	66031-1-Ig	WB (1:5000)
PDHE1α	Abcam	ab110334	WB (1:1000)
pPDHE1α	Novus	NB110-93479	WB (1:1000)
Rabbit IgG	Proteintech	30000-0-AP	WB and IF

### Isolation of human cytotrophoblast cells

Cytotrophoblast cells were isolated from term human placentas using a modified Kliman’s method as described previously^14^. In brief, aliquots of villous tissue were randomly removed from the maternal side of the placenta. The tissue was minced and digested with 0.125% trypsin (Sigma, St. Louis, MO) and 0.03% DNase I (Sigma) in Dulbecco Modified Eagle medium (DMEM, Gibco, Grand Island, NY). The dispersed placental cells were purified using a 5–65% Percoll (GE Healthcare Bio-Sciences, Uppsala, Sweden) gradient at step increments of 5%. After centrifugation, cytotrophoblasts were collected and cultured in DMEM containing 10% fetal bovine serum (FBS; Biological Industries, Israel) and 1% antibiotics (Gibco) at 37 °C in 5% CO_2_/95% air.

### Cell treatments

To study the roles of PDK4 in the regulation of carbohydrate metabolism during syncytialization, the cells were transfected with small interfering RNA (siRNA) targeting PDK4 (sense: GAUGCUCUGUGAUCAGUAUTT, antisense: AUACUGAUCACAGAGCAUCTT) (GenePharma Co., Ltd., Shanghai, China) or a eukaryotic vector GV230 expressing PDK4 (GeneChem Co., Ltd. Shanghai, China) immediately after isolation. Randomly scrambled siRNA or empty vector was transfected as a negative control respectively. Transfection was performed using an electroporator at 175 V for 5 milliseconds following a protocol as previously described^28^. The trophoblast cells were collected 48 hours after transfection. To examine the effects of hCG and the cAMP/PKA pathway in the regulation of PDK4 expression, the cells were treated after plating for 3 hours with an antibody against β-hCG (1:100, Thermo Fisher, Fremont, CA), a cAMP analogue db-cAMP (200 μM, Sigma) and a PKA inhibitor H89 (20 μM, Sigma) in DMEM containing 10% FBS for 24 hours.

### RNA sequencing

Total RNA from cultured trophoblasts before syncytialization (3 hours) and after syncytialization (48 hours) was extracted using a RNA extraction kit (OMEGA Bio-Tek, Norcross, GA) respectively. After extraction, RNA purity and integrity were determined using a NanoDrop^®^ND-2000 and an Agilent 2200 TapeStation with the following parameters: A260/A280 ratio ≥1.8, A260/A230 ratio ≥2.0 and RIN value ≥7.0. RNA-Seq libraries were prepared using a TruSeq RNA sample preparation kit (Illumina) following the manufacturer’s protocol. Sequencing of the libraries was conducted on an Illumina HiSeq™ 2500 system. A computational pipeline was used to process the RNA-seq data. Reads were aligned to human genome (UCSC hg19) using TopHat v2.1.1 with default options^29^. Differential expression analysis was performed using the Cufflinks v2.1.0^30^. Genes with RPKM ≥1 were considered expressed. The changes of PDK4 before and after syncytialization were confirmed at both mRNA and protein levels with qRT-PCR and western blotting.

### Hematoxylin staining of trophoblasts

Hematoxylin staining was conducted on cultured trophoblasts before and after syncytialization. Briefly, the cells were fixed with 4% paraformaldehyde for 10 min and then washed with PBS. The cells were stained with hematoxylin (Sigma) and mounted for microscopic examination.

### Quantitative real time PCR

The mRNA in extracted total RNA was reverse-transcribed to cDNA using a PrimeScript^®^ RT kit (TaKaRa, Dalian, China), and cDNA was applied for qRT-PCR by using power SYBR^®^ Premix Ex Taq^TM^ (TaKaRa). Housekeeping gene *ACTB* was routinely measured for normalization. The primer sequences used in qRT-PCR are illustrated in Table 2. The relative mRNA abundance was quantified with the 2^−∆∆Ct^ method.

**Table 2:** Primer sequences for qRT-PCR.

Target genes	Upstream (5′ to 3′)	Downstream (5′ to 3′)
*PDK4*	CCGTATTTCTACTCGGATGCTG	TGGCTTGGGTTTCCTGTC
*HSD11B2*	GACATGCCATATCCGTGCTT	GCTGGATGATGCTGACCTTG
*HCGB*	CGGGACATGGGCATCCAA	GCGCACATCGCGGTAGTT
*ACTB*	TGTGCAACACTTGAGTGGCT	ACTTTCTGTACTGCGGGTGG

### Western blotting

Whole cell lysate protein was prepared from the above treated cells by using ice-cold radio immunoprecipitation assay buffer (Active Motif, Carlsbad, CA) containing a complete protease inhibitor cocktail (Roche, Basel, Schweiz) and phosphatase inhibitor (Active Motif). A standard procedure of western blotting was performed as described previously^27^. Briefly, after determination of protein concentration with Bradford assay, 20 µg protein of each sample was electrophoresed in SDS-polyacrylamide gels and transferred to nitrocellulose membranes (Merck Millipore). After blocking and incubation with primary antibodies (Table 1), the membranes were incubated with appropriate secondary antibodies conjugated with horseradish peroxidase (1:5000; Proteintech). The peroxidase activities of target protein bands were detected using an enhanced chemiluminescent detection system (Merck Millipore) and visualized by using a G-Box capture system (Syngene, Cambridge, UK). The abundance of phosphorylated PDHE1α was expressed as the ratio over total PDHE1α. The abundance of PDK4 and 11β-HSD2 was expressed as the ratio over the housekeeping gene α-Tubulin.

### ATP Assay

Cellular ATP abundance was determined by using an ATP Bioluminescence Assay Kit (Beyotime, Shanghai, China) following a protocol provided by the manufacturer. Briefly, the trophoblast cells (1.5 × 10^6^ cells) before and after syncytialization as well as after manipulation of PDK4 expression were lysed with the lysis buffer provided in the kit. After centrifugation at 12,000 × g for 5 min at 4 °C, 20 μL of the supernatant was mixed with the detecting reagent (provided in the kit) to determine ATP concentrations with a tube-luminometer. The protein concentration of each sample was determined with Bradford assay. The cellular ATP level was expressed as the ratio of ATP abundance over protein concentration.

### Lactate Assay

Cellular lactate level was measured as described previously^31^. Briefly, the trophoblast cells (1.5 × 10^6^ cells) before and after syncytialization as well as after manipulation of PDK4 expression were detached from the culture plate with trypsin. After centrifugation for 10 sec at 12,000 × g, the cell pellet was collected and resuspended in 100 µL ice-cold H_2_O for 15 min. After centrifugation for 5 min at 12,000 × g, the supernatant was collected to measure lactate abundance with a Lactate Assay Kit (BioVision, Milpitas, CA). The protein concentration of each sample was determined with Bradford assay. The cellular lactate level was indicated as the ratio of lactate abundance over protein abundance.

### Statistical analysis

All data are reported as mean ± SEM. The number for each experiment indicates repeated experiments using trophoblast cells from different placentas. Paired Student’s t-test or one-way ANOVA test followed by the Student-Newman-Keuls test was used where appropriate to assess statistical significant differences. Significance was set at P < 0.05.

### Data availability statement

All data generated or analyzed during this study are included in this published article and its Supplementary Information files.

## Electronic supplementary material

Dataset 1

## References

[1] Gray LR, Tompkins SC, Taylor EB (2014). Regulation of pyruvate metabolism and human disease. Cellular and molecular life sciences: CMLS..

[2] Vander Heiden MG, Cantley LC, Thompson CB (2009). Understanding the Warburg effect: the metabolic requirements of cell proliferation. Science..

[3] Coller HA (2014). Is cancer a metabolic disease?. Am J Pathol..

[4] Folmes CD, Dzeja PP, Nelson TJ, Terzic A (2012). Metabolic plasticity in stem cell homeostasis and differentiation. Cell Stem Cell..

[5] Kaplon J (2013). A key role for mitochondrial gatekeeper pyruvate dehydrogenase in oncogene-induced senescence. Nature..

[6] Korotchkina LG, Patel MS (2001). Site specificity of four pyruvate dehydrogenase kinase isoenzymes toward the three phosphorylation sites of human pyruvate dehydrogenase. J Biol Chem..

[7] Takubo K (2013). Regulation of glycolysis by Pdk functions as a metabolic checkpoint for cell cycle quiescence in hematopoietic stem cells. Cell Stem Cell..

[8] Ferretti C (2007). Molecular circuits shared by placental and cancer cells, and their implications in the proliferative, invasive and migratory capacities of trophoblasts. Human reproduction update..

[9] Maloyan A, Mele J, Muralimanohara B, Myatt L (2012). Measurement of mitochondrial respiration in trophoblast culture. Placenta..

[10] Kliman HJ (1986). Purification, characterization, and *in vitro* differentiation of cytotrophoblasts from human term placentae. Endocrinology..

[11] Aronow BJ, Richardson BD, Handwerger S (2001). Microarray analysis of trophoblast differentiation: gene expression reprogramming in key gene function categories. Physiol Genomics..

[12] Mori M (2007). The cytotrophoblast layer of human chorionic villi becomes thinner but maintains its structural integrity during gestation. Biology of reproduction..

[13] Huppertz B, Borges M (2008). Placenta trophoblast fusion. Methods Mol Biol..

[14] Li JN (2011). The Sp1 transcription factor is crucial for the expression of 11beta-hydroxysteroid dehydrogenase type 2 in human placental trophoblasts. J Clin Endocrinol Metab..

[15] Costa MA (2016). Scrutinising the regulators of syncytialization and their expression in pregnancy-related conditions. Mol Cell Endocrinol..

[16] Weedon-Fekjaer, M. S. & Tasken, K. Review: Spatiotemporal dynamics of hCG/cAMP signaling and regulation of placental function. Placenta. **33** Suppl, S87–91, (2012). 10.1016/j.placenta.2011.11.003 22103973

[17] Acin-Perez R (2009). Cyclic AMP produced inside mitochondria regulates oxidative phosphorylation. Cell metabolism..

[18] Ravnskjaer K, Madiraju A, Montminy M (2016). Role of the cAMP Pathway in Glucose and Lipid Metabolism. Handbook of experimental pharmacology..

[19] Palorini R (2016). Protein Kinase A Activation Promotes Cancer Cell Resistance to Glucose Starvation and Anoikis. PLoS genetics..

[20] Pagliarini DJ, Dixon JE (2006). Mitochondrial modulation: reversible phosphorylation takes center stage?. Trends in biochemical sciences..

[21] Zhou Q, Lam PY, Han D, Cadenas E (2008). c-Jun N-terminal kinase regulates mitochondrial bioenergetics by modulating pyruvate dehydrogenase activity in primary cortical neurons. Journal of neurochemistry..

[22] Ramalho-Santos J (2009). Mitochondrial functionality in reproduction: from gonads and gametes to embryos and embryonic stem cells. Human reproduction update..

[23] Zhang J (2011). UCP2 regulates energy metabolism and differentiation potential of human pluripotent stem cells. EMBO J..

[24] Vazquez-Martin A (2013). The mitochondrial H(+)-ATP synthase and the lipogenic switch: new core components of metabolic reprogramming in induced pluripotent stem (iPS) cells. Cell Cycle..

[25] Xie, Y. *et al*. Hypoxic stress induces, but cannot sustain trophoblast stem cell differentiation to labyrinthine placenta due to mitochondrial insufficiency. Stem Cell Res, (2014). 10.1016/j.scr.2014.07.007 PMC4253717 25239494

[26] Martinez, F., Kiriakidou, M. & Strauss, J. F., 3rd. Structural and functional changes in mitochondria associated with trophoblast differentiation: methods to isolate enriched preparations of syncytiotrophoblast mitochondria. Endocrinology. **138**, 2172–2183, (1997). 10.1210/endo.138.5.5133 9112417

[27] Zuo RJ (2015). Warburg-like Glycolysis and Lactate Shuttle in Mouse Decidua during Early Pregnancy. J Biol Chem..

[28] Wang W (2015). Phosphorylation of STAT3 mediates the induction of cyclooxygenase-2 by cortisol in the human amnion at parturition. Science signaling..

[29] Trapnell C, Pachter L, Salzberg SL (2009). TopHat: discovering splice junctions with RNA-Seq. Bioinformatics..

[30] Trapnell C (2010). Transcript assembly and quantification by RNA-Seq reveals unannotated transcripts and isoform switching during cell differentiation. Nature biotechnology..

[31] Doherty JR (2014). Blocking lactate export by inhibiting the Myc target MCT1 Disables glycolysis and glutathione synthesis. Cancer research..

